# The Challenging *in silico* Description of Carbon Monoxide Oxidation as Catalyzed by Molybdenum-Copper CO Dehydrogenase

**DOI:** 10.3389/fchem.2018.00630

**Published:** 2019-01-09

**Authors:** Anna Rovaletti, Maurizio Bruschi, Giorgio Moro, Ugo Cosentino, Claudio Greco

**Affiliations:** ^1^Dipartimento di Scienze dell'Ambiente e della Terra, Università Degli Studi di Milano-Bicocca, Milan, Italy; ^2^Dipartimento di Biotecnologie e Bioscienze, Università Degli Studi di Milano-Bicocca, Milan, Italy

**Keywords:** molybdenum, copper, dehydrogenase, DFT, carbon monoxide

## Abstract

Carbon monoxide (CO) is a highly toxic gas to many living organisms. However, some microorganisms are able to use this molecule as the sole source of carbon and energy. Soil bacteria such as the aerobic *Oligotropha carboxidovorans* are responsible for the annual removal of about 2*x*10^8^ tons of CO from the atmosphere. Detoxification through oxidation of CO to CO_2_ is enabled by the MoCu-dependent CO-dehydrogenase enzyme (MoCu-CODH) which—differently from other enzyme classes with similar function—retains its catalytic activity in the presence of atmospheric O_2_. In the last few years, targeted advancements have been described in the field of bioengineering and biomimetics, which is functional for future technological exploitation of the catalytic properties of MoCu-CODH and for the reproduction of its reactivity in synthetic complexes. Notably, a growing interest for the quantum chemical investigation of this enzyme has recently also emerged. This mini-review compiles the current knowledge of the MoCu-CODH catalytic cycle, with a specific focus on the outcomes of theoretical studies on this enzyme class. Rather controversial aspects from different theoretical studies will be highlighted, thus illustrating the challenges posed by this system as far as the application of density functional theory and hybrid quantum-classical methods are concerned.

Carbon monoxide (CO) is a fatal gas to many living organisms as well as an indirect greenhouse gas in the atmosphere (Liu et al., 2018). Global CO emissions derive both from anthropogenic and natural sources (Choi et al., 2017). One of the main sinks of atmospheric CO is constituted by the soil, in which it is consumed in large amounts by microbial oxidation (Liu et al., 2018). One example of these important soil microorganisms is represented by the aerobic bacteria *Oligotropha carboxidovorans*. The latter is able to grow using CO as its sole source of carbon and energy (Hille et al., 2015). This metabolism is ascribed to the air-stable Mo/Cu-dependent CO dehydrogenase (MoCu-CODH) enzyme that catalyzes the oxidation of CO to CO_2_ (Zhang et al., 2010).

This enzyme contains a unique active site composed by a molybdenum/copper bimetallic center (see Figure 1A). The molybdenum ion is found in a square-pyramidal geometry with one apical oxo ligand, a dithiolene ligand from the molybdopterin cytosine dinucleotide (MCD) cofactor, an equatorial oxo ligand and a sulfido ligand. The latter bridges to the copper center, which links the active site to the protein matrix by coordinating the sulfur atom of Cys388. Moreover, Cu is also coordinated to a weakly bound water molecule (Gnida et al., 2003; Rokhsana et al., 2016), and can coordinate not only CO (i.e., the physiologic substrate) but also H_2_. In fact, the MoCu-CODH enzyme has the ability to catalyze dihydrogen oxidation, even though such hydrogenase activity is rather low (Santiago and Meyer, 1996; Wilcoxen and Hille, 2013).

**Figure 1:**
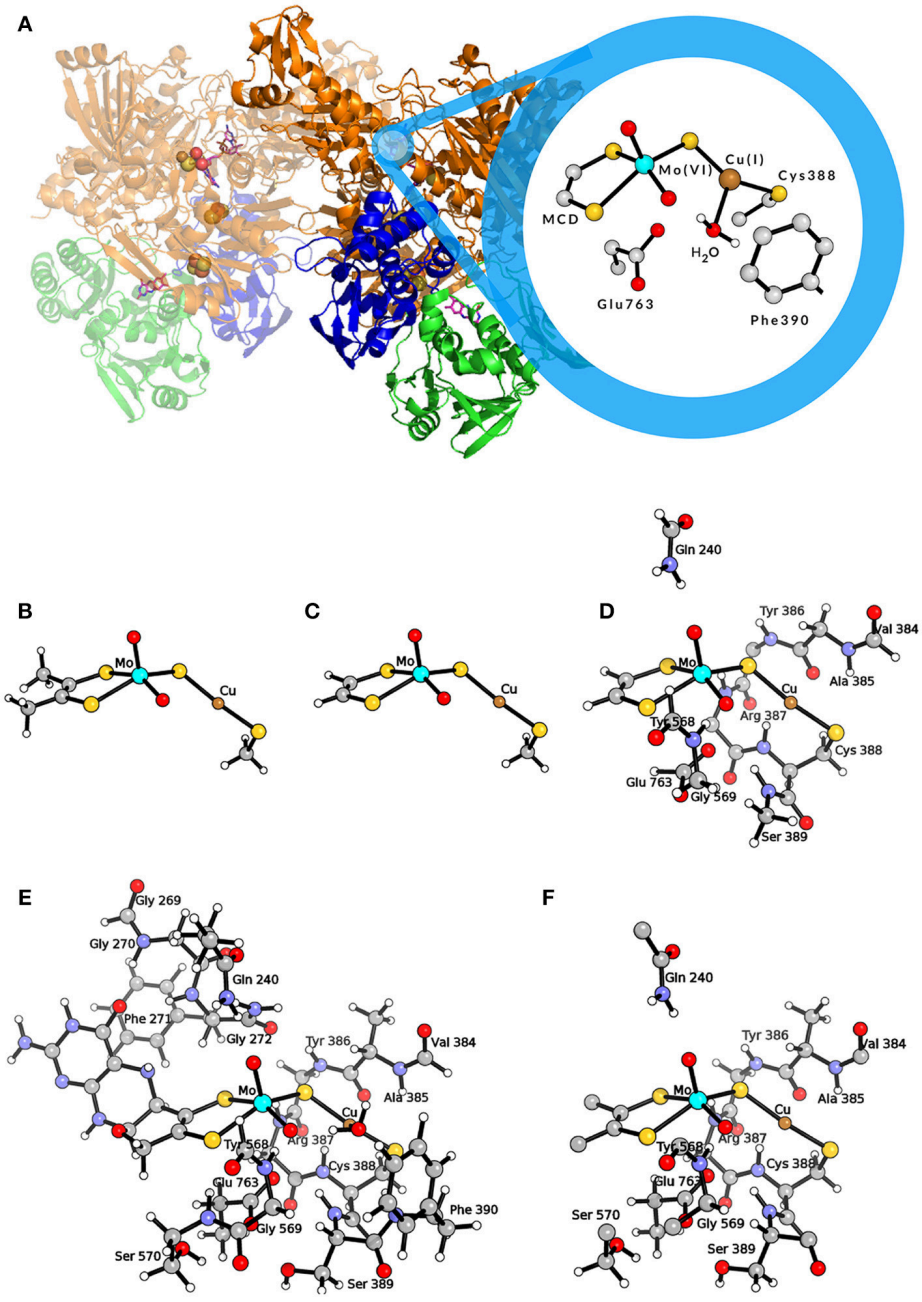
**(A)** Representation of the MoCu-CODH enzyme and of the active site in the Mo(VI)Cu(I) resting state. **(B)** QM model used by Hofmann et al. (2005) and by Stein and Kirk (2014); **(C)** QM model used by Siegbahn and Shestakov (2005) and by Breglia et al. (2017); **(D)** larger QM model used by Siegbahn and Shestakov (2005); **(E)** QM model used by Rokhsana et al. (2016); **(F)** QM region of the hybrid QM/MM model used by Xu and Hirao (2018). Color code of atoms in the active site: cyan, molybdenum; orange, copper; yellow, sulfur; red, oxygen; gray, carbon; white, hydrogen.

The protonation state of the active site has been object of debate. In fact, experimental X-ray diffraction (XRD) results were interpreted as indicative of a Mo(=O)OH state both for the oxidized and the reduced forms of the enzyme (Dobbek et al., 2002). Differently, extended X-ray absorption fine structure (EXAFS) spectroscopy suggested the presence of a Mo^*VI*^(=O)O unit and a Mo^*IV*^(=O)OH_2_ unit for the oxidized and reduced states, respectively (Gnida et al., 2003). Recent data in support of the EXAFS-based hypothesis came from electron paramagnetic resonance (EPR) analysis of the Mo^*V*^ analog and from theoretical calculations (Zhang et al., 2010; Rokhsana et al., 2016). As far as the oxidation state of the Cu ion is concerned, it maintains the +1 state throughout the enzymatic catalytic cycle (Dobbek et al., 2002; Gnida et al., 2003). In fact, CO oxidation occurs directly at the Cu center (Shanmugam et al., 2013), and the two-electron transfer to Mo at each catalytic cycle is allowed by the highly delocalized nature of the Mo(μ-S)Cu unit (Gourlay et al., 2006). Key second-sphere amino acid residues are conserved in MoCu-CODH enzymes and in homologues with different activity, i.e., xanthine oxidases (Hille, 2013). In particular, a conserved glutamate residue (Glu763) is found in proximity to the equatorial oxo ligand of Mo and is considered to act as a base to facilitate deprotonation events (Wilcoxen and Hille, 2013; Hille et al., 2015). Moreover, the aromatic ring of a phenylalanine residue (Phe390), located in front of the Cu^*I*^ ion, is thought to have relevance for the correct positioning of the substrate within the active site (Rokhsana et al., 2016).

Carbon monoxide (|C≡O) is expected to show similar reactivity with respect to the isoelectronic isocyanide (|C≡N-R) species. They share the presence of non-bonding electron pairs in the *sp* orbital of the terminal carbon atom and a triple bond between the latter and a more electronegative atom. Indeed, Dobbek et al. reported the inhibitory activity of *n*-butylisocyanide, |C≡N-(CH_2_)_3_CH_3_, toward the oxidized MoCu-CODH (Dobbek et al., 2002). In the same study, the corresponding crystallographic structure of the inhibited enzyme was determined (PDB ID: 1N62). The resulting inactive complex is characterized by a thiocarbamate geometry in which the isocyanide group forms covalent bonds with the μ-sulfido ligand, the equatorial oxygen of Mo and the Cu atom, while the alkyl chain of *n*-butylisocyanide extends into the hydrophobic interior of the substrate channel.

The features of the crystal structure of the *n*-butylisocyanide-bound state prompted Dobbek and coworkers to advance the first hypothesis ever proposed for the MoCu-CODH catalytic mechanism (see Figure 2A) (Dobbek et al., 2002). The latter involves the formation of a thiocarbonate-like intermediate—analogous to the thiocarbamate derivative formed during the aforementioned inhibition—after the CO substrate accesses the oxidized active site. Such thiocarbonate species would be characterized by the insertion of CO between copper, the μ-sulfido ligand and the equatorial oxo ligand of the Mo atom. Taking inspiration from such a mechanistic hypothesis for CO-oxidation catalysis, the enzymatic mechanism has been subsequently studied with computational methods by several groups, thus giving birth to a debate that is still ongoing (*vide infra*).

**Figure 2:**
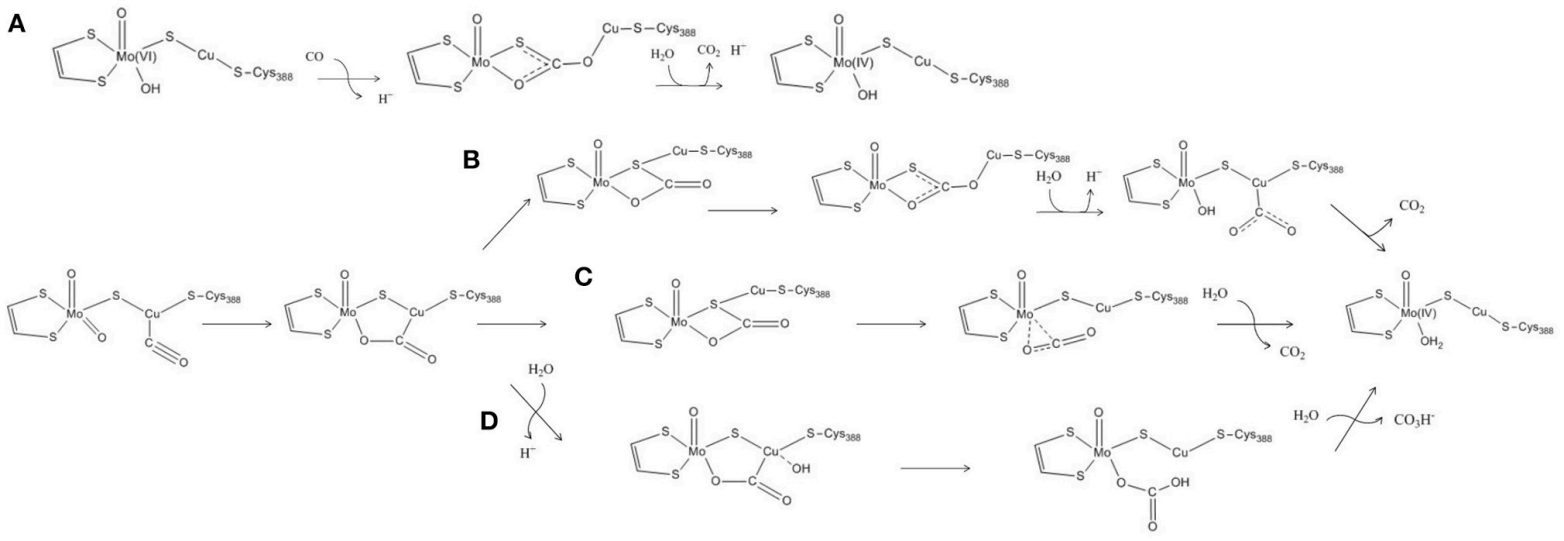
Proposed reaction mechanisms for the oxidation of CO by means of MoCu-CODH. **(A)** Reaction mechanism proposed by Dobbek's experimental group. **(B–D)** Reaction mechanisms proposed by Siegbahn and Shestakov, by Hofmann et al. and by Stein and Kirk, on the basis of their respective computational studies.

Recently, it has been reported that rather bulky thiol molecules—e.g., L-cysteine, coenzyme A or glutathione—can reach the bimetallic active site (Kreß et al., 2014). They cause a reversible inactivation of the enzymatic activity, competing with the substrates for the same position at the Cu^*I*^ center.

In the following two sections of this manuscript we aim at reviewing the theoretical studies that have been published on MoCu-CODH using quantum mechanical (QM) and hybrid quantum mechanical/molecular mechanical (QM/MM) approaches. In doing so, we will pay special attention to the controversies and the challenges that have emerged. Moreover, promising future developments in the theoretical description of this system will be proposed in the concluding section.

## 1. Small- and Medium-Sized QM-Cluster Models

The first theoretical investigations of the catalytic activity of MoCu-CODH were carried out independently by two theoretical groups in 2005 (Hofmann et al., 2005; Siegbahn and Shestakov, 2005). To model the enzymatic active site, Hofmann et al. used a small cluster of 24 atoms representing the two transition metals and their first coordination spheres (see Figure 1B) (Hofmann et al., 2005), whereas Siegbahn and Shestakov performed calculations with two different models, a small one of about 20 atoms and a bigger one composed of about 70 atoms (see Figures 1C,D). In the latter, some residues belonging to the second coordination spheres of the metals were explicitly included (Siegbahn and Shestakov, 2005). The hybrid density functional B3LYP (Lee et al., 1988; Becke, 1993) was employed in both cases to optimize the geometries and compute relative energies of intermediates along the putative catalytic cycles. For geometry optimizations, Hofmann and coworkers adopted the Lanl2DZ (Dunning and Hay, 1976; Hay and Wadt, 1985a,b; Wadt and Hay, 1985) effective core basis set with additional d-type functions on S atoms. This was followed by single point energy calculations using the SDD (Dunning, 1970; Dunning and Hay, 1977; Dolg et al., 1987) basis set augmented by d-type polarization functions on all non-hydrogen, non-metal atoms. The basis sets employed by Siegbahn and Shestakov were lacvp and lacv3p*—with ECP for Mo, Cu, and S atoms—for geometry optimizations and energy calculations, respectively. In both studies, the protein matrix surrounding the active site was modeled by a continuum dielectric with ϵ = 4 (Eckert and Klamt, 2002; Cossi et al., 2003).

A comparative analysis of the results coming from two such early investigations evidences a significant variability of the results obtained as a function of the adopted level of theory, within models of the same size. In particular, the calculated energy differences showed significant dependency on the basis sets used, with deviations up to 21 kJ/mol in the case of intermediates involved in formation of the new C–O bond, a key step in the catalytic process. Interestingly, previous studies of oxygen-atom transfer (OAT) reactions involving Mo-complexes (Li et al., 2013) and other transition-metal-containing systems (Hu and Chen, 2015; Li et al., 2015) also evidenced the strong basis set effect on computed energy differences along the reactive paths.

Notwithstanding the shortcomings deriving from the choice of basis sets, both Siegbahn, Shestakov and their respective coworkers evidenced a surprisingly high stability for the thiocarbonate intermediate. The presence of such a deep minimum on the energy landscape pertaining to the reaction mechanism was interpreted differently by the two groups. Siegbahn and Shestakov in particular proposed that the thiocarbonate derivative represents an intermediate of the CO-oxidation mechanism. However, in the proposed mechanism the barrier for the release of the CO_2_ product was estimated to be rather high, as it would require the insertion of a water molecule which was reported not to be a facile step (see Figure 2B). Differently, Hofmann and coworkers raised the possibility that the thiocarbonate adduct lies outside the catalytic cycle, in a deep potential energy well that would effectively slow down enzymatic activity (see Figure 2C). These authors further proposed that the constrains imposed by the protein matrix could prevent formation of such a stable off-path adduct, a hypothesis that— however—was later discarded as a result of a theoretical study focused on this topic (Siegbahn, 2011).

In a more recent theoretical study, a different mechanism for the oxidation of CO by the MoCu-CODH enzyme was proposed (see Figure 2D) (Stein and Kirk, 2014). Using a cluster model analogous to the one previously employed by Hofmann and coworkers (see Figure 1B), at the PBE/TZP (Perdew et al., 1996; Ernzerhof and Scuseria, 1999) level of theory and including continuum dielectric contributions (ϵ = hexane) (Klamt and Schüürmann, 1993), Stein and Kirk proposed that the stable thiocarbonate intermediate formation could be bypassed by evolving bicarbonate as a final product rather than CO_2_. Bicarbonate formation would proceed via nucleophilic attack of a copper-activated water molecule on the C atom of the metal-bound CO_2_. However, such a picture is at odds with recent experimental studies, which appear to exclude the possibility of forming a bicarbonate complex during catalysis (Dingwall et al., 2016).

Breglia and coworkers published the most recent theoretical study of MoCu-CODH, in which only the first shell coordination spheres were included in a QM model (see Figure 1C) (Breglia et al., 2017). Such a study mainly regards the hydrogenase activity of the enzyme and includes a comparative analysis of the binding reactions of the physiologic substrate—i.e., CO—and of dihydrogen to the Cu ion. Similarly to previous studies on H_2_- and CO-binding enzyme models in which a pure functional was used in conjuction with triple-zeta bases (Greco et al., 2015; Rovaletti and Greco, 2018), geometry optimizations and energy calculations were carried out in vacuo at BP86/def2-TZVP level (Perdew, 1986; Becke, 1988; Weigend and Ahlrichs, 2005). As far as the energetics of CO binding is concerned, the computed ΔE was as negative as −64 kJ/mol. A comparison of the latter value with the results of corresponding calculations previously performed at different levels of theory for the same reaction by Siegbahn, Hofmann and their respective coworkers, evidences significant discrepancies (ΔΔE = 50 and 31 kJ/mol, respectively). Actually, the occurrence of such large differences comes with little surprise, given the well-known shortcomings in binding energy calculations using quantum chemical models of coordination complexes and their ligands (Husch et al., 2018).

## 2. Large QM-Cluster Models and Hybrid Models

The importance of extending the dimension of the bimetallic active site model and systematically accounting for the effects of the second-sphere residues on energetics was evidenced in a recent theoretical work by Rokhsana and coworkers (Rokhsana et al., 2016). In fact, a large-size cluster model of around 180 atoms (see Figure 1E) turned out to be required for a fully satisfactory reproduction of experimentally determined structural parameters. The same held true for the evaluation of the most plausible protonation states of the Mo/Cu core, which was done taking into account key geometric features of the enzyme crystal structure and redox potential measurements available in literature. The authors were able to assess in particular the protonation state of the equatorial oxo ligand of Mo, at the different redox states attained during catalysis. As for the adopted level of theory, Rokhsana et al. employed the def2-TZVP basis set for all elements, apart from H and C atoms, for which the def2-SVP basis set was used (Weigend and Ahlrichs, 2005). The protein environment was modeled by a continuum dielectric with ϵ = 4 (Klamt and Schüürmann, 1993). The BP86 and B3LYP density functionals were used for geometry optimizations and energy evaluation, respectively.

In a subsequent study, explicit consideration of the whole protein environment was achieved by means of a hybrid quantum mechanics/molecular mechanics (QM/MM) approach (Xu and Hirao, 2018). In the work of Xu and Hirao, the active site was described by a QM region of 89 atoms (see Figure 1F) using the B3LYP functional. During geometry optimization, the SDD effective core potential basis set was employed to represent the transition metal ions, whereas the 6-31G* basis set was adopted for all the other atoms (Dolg et al., 1987; Andrae et al., 1990). Single-point energy calculations were carried out using the larger def2-TZVP basis set. For the molecular mechanics calculations, the AMBER03 force field was employed (Duan et al., 2003). Moreover, the Grimme's D3-correction with Becke−Johnson damping [D3(BJ)] was taken into account in the calculations (Grimme et al., 2011). The previously proposed catalytic mechanisms that involve the formation of the thiocarbonate species were re-investigated at such a level of theory. According to Xu and Hirao's study, the S-C-bound adduct would be formed along the reaction pathway as previously suggested (Dobbek et al., 2002; Siegbahn and Shestakov, 2005). However, based on the novel QM/MM results, the thiocarbonate species thus formed would not be as stable as previously proposed. It has to be remarked that the thiocarbonate intermediate was not found to be directly linked to the transition state for CO_2_ releasing. In fact, it was proposed that— after thiocarbonate formation—the reaction needs to follow a reverse process to productively proceed toward CO_2_ evolution. Notably, the overall barrier for the proposed catalytic mechanism was found to be low (in the order of 50 kJ/mol). In the same study, Xu and Hirao also carried out purely QM calculations with a QM-cluster of the same size of the quantum-mechanical region of their hybrid model, and compared the obtained results with those coming from QM/MM modeling. Such a comparison evidenced that the protein environment is not involved in modulating the kinetic barrier associated with the investigated catalytic mechanism. However, it was found that the protein matrix plays an important role in the stabilization of the CO_2_-released state. Finally, it was also reported that the inclusion of dispersive corrections lowers by 15 kJ/mol the activation barrier of the product-releasing step, in line with what was expected for the modeling of a bimolecular reaction step.

## Concluding Remarks

Over the last fifteen years, the theoretical investigation of the CO oxidation mechanism by MoCu-CODH has given rise to a debate, the essentials of which are centered on the possible occurrence and on the role of a thiocarbonate catalytic intermediate. In the above sections, we have reported key details of the various computational studies published to date, and we are now in the condition to present a more general outlook on the state of the art regarding MoCu-CODH.

The early studies by Siegbahn, Hofmann, and their respective coworkers evidenced that the thiocarbonate intermediate would occupy a deep well in the energy profiles pertaining to the investigated reaction mechanisms. However, the kinetic barriers they computed for CO_2_ evolution were at least 30 kJ/mol higher than the recently determined experimental counterpart (Zhang et al., 2010). In part, this picture depends on the neglect of dispersive corrections: their inclusion became a standard possibility only after the publication of the mentioned study (Siegbahn, 2011).

Results more compatible with the experimental evidence of a kinetic barrier of around 50 kJ/mol were obtained by Xu and Hirao, who exploited a larger QM-cluster model with the explicit inclusion of most of the second-sphere coordination environment, along with employment of large basis sets and dispersion corrections (Xu and Hirao, 2018). It is noticeable that, according to Xu and Hirao's results, the thiocarbonate species still appears to behave as a thermodynamic sink. Even though not a very deep one, such a sink would effectively hamper the advancement along the proposed path toward products, a rather unusual role for a species formed during an enzymatic process.

All the catalytic mechanisms proposed in the theoretical studies reviewed here focus on the possibility that the Mo-bound equatorial oxo ligand performs the nucleophilic attack on the activated CO substrate bound to the Cu ion. This is in line with what has been suggested in the case of the catalytic mechanism of the homologous xanthine oxidases enzymes. However, a variant with respect to such a picture has been recently proposed, in which an activated water molecule would play the role of nucleophile (Hille et al., 2015). Notably, to the best of our knowledge this alternative mechanism has not yet been investigated by QM or hybrid QM/MM studies.

Future theoretical studies on this (and other) putative catalytic mechanism will possibly face the challenge associated with rather pronounced fluctuations of computed energy differences as a function of the adopted level of theory. As far as the application of density functional theory is concerned, extensive benchmarking could in principle help to improve the theoretical predictions. In this regard, the available experimental data on enzyme inhibition— by thiols in particular—could represent a useful dataset, keeping in mind that the reproduction of binding energies in the case of bulky thiols might require extensive protein matrix phase space sampling in the case of QM/MM studies. High level *ab initio* methods are a viable—though still challenging—alternative for providing reliable results. In fact, thanks to recent methodological developments, the treatment of relatively large bimetallic systems has been shown to be computationally affordable with multiconfigurational post-Hartree-Fock approaches (Phung et al., 2016, 2018; Dong et al., 2017).

The relevance of MoCu-CODH as an inspiring system for future biomimetic and bioengineering applications is currently growing. This is due not only to the relevance of the reactions it catalyzes, but also to its resistance to atmospheric O_2_ exposure—a rare feature in the case of enzymes expressing carbon monoxide dehydrogenase and hydrogenase activities (Choi et al., 2017; Gourlay et al., 2018; Groysman et al., 2018). Notably, the recent establishment of a functional heterologous expression system for the MoCu-CODH enzyme (Kaufmann et al., 2018) together with developments in the computational chemistry field will hopefully boost the positive feedback among biochemical, biomimetic and quantum chemical studies, opening new perspectives for a deeper understanding of this interesting metalloenzyme.

## Author Contributions

All authors listed have made a substantial, direct and intellectual contribution to the work, and approved it for publication.

### Conflict of Interest Statement

The authors declare that the research was conducted in the absence of any commercial or financial relationships that could be construed as a potential conflict of interest.
